# Personality and Attachment Patterns in Patients with Psychogenic Non-Epileptic Seizures in Saudi Arabia

**DOI:** 10.3390/medicina60121926

**Published:** 2024-11-23

**Authors:** Nadia Al-Tamimi, Majed Al-Hameed, Mohammed M. J. Alqahtani, Mohammad Uzair, Shahid Bashir, Haythum Tayeb, Ahmed Abu-Zaid

**Affiliations:** 1 Department of Mental Health, National Neuro-Science Center, King Fahad Medical City, Riyadh 12231, Saudi Arabia; 2 Neuroscience Centre, King Faisal Specialist Hospital and Research Center, Riyadh 12713, Saudi Arabia; 3 Department of Bioengineering, King Fahd University of Petroleum and Minerals, Dhahran 31261, Saudi Arabia; 4 Neuroscience Centre, King Fahad Specialist Hospital Dammam, Dammam 32253, Saudi Arabia; 5 The Mind and Brain Studies Initiative, The Neuroscience Research Unit, Faculty of Medicine, King Abdulaziz University, Jeddah 22254, Saudi Arabia; 6 Department of Biochemistry and Molecular Medicine, College of Medicine, Alfaisal University, Riyadh 11533, Saudi Arabia

**Keywords:** psychogenic non-epileptic seizures (PNES), personality patterns, attachment styles, psychological profiles, epilepsy

## Abstract

*Background and Objectives:* The purpose of this study was to investigate personality and relationship patterns in patients with psychogenic non-epileptic seizures (PNES) and compare them to patients with epilepsy and healthy controls. *Materials and Methods:* A total of 68 participants were recruited (mean age = 29.8 ± 9.4 years), including 25 (36.2%) with PNES. The assessment was conducted using the Relationship Questionnaire (RQ), Big Five Inventory (BFI), Relationship Assessment Scale (RAS), Satisfaction with Life Scale (SWLS), and Conflict Behavior Scale (CBS). *Results:* The IQ of patients with PNES (88.8 ± 13.6) was lower compared to healthy controls (103.5 ± 28.0) but higher than epilepsy patients (84.6 ± 12.9). There were no significant differences between PNES patients and either patients with epilepsy or healthy controls in terms of security, fearfulness, preoccupation, or dismissiveness based on RQ subscale scores. PNES patients tended to be less satisfied (RAS total score, *p* = 0.10), but did not differ on overall life quality (on SWLS) compared to epilepsy patients and healthy individuals. There were no significant differences in the scores for different attachment styles (secure, fearful, preoccupied, dismissive) among the groups (*p* > 0.05). Significant differences were found in agreeableness (*p* = 0.017) and openness (*p* = 0.009) among the groups. The PNES group exhibits higher scores in Negative—Own (*p* = 0.009), Positive—Own (*p* = 0.011), Negative—Partner (*p* = 0.011), and Positive—Partner (*p* = 0.028) compared to epilepsy and healthy individuals. No significant differences observed in the Abusive—Own and Abusive—Partner scores (*p* > 0.05). *Conclusions:* In conclusion, this study highlights distinct personality traits and relationship patterns in patients with psychogenic non-epileptic seizures (PNES) compared to epilepsy patients and healthy controls, emphasizing the need for targeted interventions to address these psychological nuances effectively.

## 1. Introduction

Psychogenic non-epileptic seizures (PNES) are a subtype of functional neurologic symptom disorder (FND), characterized by seizure-like episodes without associated epileptiform changes on electroencephalogram (EEG) recordings [1,2,3]. The pathophysiology of PNES remains incompletely understood. Despite the absence of epileptiform EEG changes, neuroimaging studies have identified evidence of neural circuit dysfunction that may contribute to PNES. However, psychopathology, personality patterns, attachment styles, and early-life trauma are considered central components of the pathophysiology of PNES [4]. Sexual and physical abuse are identified as common risk factors for PNES in Western populations [5,6]. In addition, various psychosocial stressors, such as rape, relationship difficulties, divorce, job loss, death of loved ones, physical illness, and natural disasters may predispose susceptible individuals to PNES [7]. Attachment theory provides a framework for understanding the development and maintenance of pseudo-seizures and the influence of childhood abuse or neglect on these processes [8]. Individuals with secure attachments tend to have healthier coping mechanisms and emotional regulation, potentially reducing the risk of developing PNES, while those with fearful or preoccupied attachment styles may experience higher levels of stress and emotional dysregulation, increasing vulnerability to PNES. In contrast, individuals with a dismissive attachment style might suppress emotional distress, leading to somatic expressions like PNES as a way to cope with unprocessed feelings [9].

The epidemiological understanding of PNES extends beyond developed Western countries and is recognized as a global phenomenon. While previous studies on PNES have predominantly focused on Western countries, PNES is acknowledged as a cultural phenomenon in various cultures worldwide. Limited research has also been conducted in other countries with distinct sociodemographic and religious backgrounds, such as Argentina, Brazil, China, India, Turkey, Croatia, South Africa, etc. [10,11]. However, there remains a scarcity of data on PNES in Middle Eastern countries.

Therefore, this study aims to identify various psychological indicators related to pseudo-seizure patients in the Saudi Arabian population. This will be achieved by utilizing a comprehensive battery of tests assessing personality traits, relationship status, and overall life satisfaction.

## 2. Materials and Methods

The local Institutional Ethics Committee at King Fahad Medical City, Riyadh, Saudi Arabia, approved this study, and participants provided written informed consent. The research design employed was a cross-sectional study. Participants were recruited from the epilepsy-monitoring units (EMUs) of two tertiary care centers. Individuals meeting the inclusion criteria were included in the study. The inclusion criteria were the following: (1) age between 18 and 60 years; (2) Saudi nationality; and (3) confirmed diagnosis of epilepsy or PNES. In our study, the diagnosis of epileptic seizures and PNES was confirmed through a combination of clinical assessments, neurophysiological testing, and, in some cases, psychological evaluation. For epileptic seizures, diagnosis was based on a clear history of seizures, characteristic clinical features, and the confirmation of abnormal EEG activity during seizures. For PNES, diagnosis was primarily reached using video–EEG monitoring, where normal EEG patterns during seizure-like events ruled out epilepsy. Additionally, patients with PNES were evaluated for underlying psychological factors through clinical interviews and psychiatric assessment. Patients with combined epilepsy and PNES; individuals with other established psychiatric diagnoses such as schizophrenia, bipolar affective disorder, or intellectual disabilities; and those who were illiterate (unable to read or write) were excluded.

Patients were successively approached until the target sample size of each category was achieved. Healthy controls were recruited from hospital employees not undergoing monitoring in the EMU or mental health clinics. Family and friends accompanying patients for other medical specialties were also considered potential healthy controls.

The determination of the target sample size for patients with epilepsy, PNES, and healthy controls was based on prior studies and involved a power calculation aimed at detecting a moderate effect size (Cohen’s D = 0.5).

A purposefully designed questionnaire was used to collect relevant sociodemographic and clinical data. Participants were asked to provide details on age, gender, marital status, and qualification.

A battery of psychological tests was used to determine and identify the factors and characteristics of pseudo-seizure patients and correlate them with that of epilepsy patients, as well as controls. The psychodiagnostics battery used in this study to assess personality and attachment patterns included the Relationship Questionnaire (RQ), Big Five Inventory (BFI), Relationship Assessment Scale (RAS), Satisfaction with Life Scale (SWLS), and Conflict Behavior Scale (CBS).

The Relationship Questionnaire (RQ) consists of questions that load on four attachment styles, namely secure, fearful, preoccupied, and dismissive. RQ assesses adult attachment styles to the participant’s spouse. The second tool used was the Big Five Inventory (BFI). This battery consists of 44 items. It assesses personality traits, including extraversion, agreeableness, conscientiousness, neuroticism, and openness [12]. However, in this study, we used the BFI-K scale [13] with a few amendments, i.e., one item related to artistic skills (i.e., acting or playing) was excluded because these are not common activities in Saudi Arabia. Extraversion (or extroversion) refers to a personality trait characterized by excitability, sociability, talkativeness, assertiveness, and emotional expressiveness, where being around others energizes and excites the individual. Individuals with a total score of items 1, 2, 3, and 4 and a moderate score of 6 and above, up to the maximum score of 12, were considered more extroverted). Agreeableness entails traits such as trust, selflessness, kindness, and affection, with high scores (>8) indicating no agreeableness (cooperative behavior) and low scores (<8) implying more agreeableness. Conscientiousness is marked by high levels of thoughtfulness, self-control, and goal-directed behaviors, with highly conscientious people (score 9–16) being organized, detail-oriented, and considerate of others. Neuroticism reflects emotional instability, with higher scores (9–16) indicating greater emotional distress. Openness describes a person with diverse interests, curiosity about the world, and a tendency to seek new experiences, displaying creativity (score ≥ 10 indicates more openness).

The Relationship Assessment Scale (RAS) contains 7 items, of which 4 items aimed at assessing passion, freedom, and independence were added to increase the scope of the assessment scale [14]. The newly added items were inserted at the end of the scale, becoming items 8, 9, 10, and 11. An example of a newly added item is “I’m happy with the amount of freedom and independence I have in my marriage.” The answer format was a 5-point Likert scale ranging from 1 (low satisfaction) to 5 (high satisfaction). The maximum score was 98 (very satisfied), and a score of 49 was considered moderate. The RAS questionnaire was administered to married couples only, in which one of the partners was diagnosed with either epilepsy or pseudo-seizures, and among the control group.

The Satisfaction with Life Scale (SWLS) consists of 5 items that measure the participants’ evaluation of life satisfaction through their cognitive judgment of global life satisfaction [15]. The global-level items in this scale include statements such as “In most ways, my life is close to my ideal”. The answer format used by participants to respond to this questionnaire was the 5-point Likert agreement scale. The maximum is 25 (very satisfied), the average is 13, and below 13 is less satisfied. This scale was used to investigate whether people diagnosed with epilepsy perceive their life satisfaction to be reduced because of the diagnosis [16].

The Conflict Behavior Scale (CBS) combines the Rusbult Problem Solving Scale [17] and the Conflict Tactics Scale [18] to measure participants’ responses to relationship problems. The Rusbult Problem Solving scale assesses behaviors characterized as exit, voice, loyalty, and neglect, with voice and loyalty considered constructive and exit and neglect considered destructive [17]. The Conflict Tactics Scale measures explicit actions during conflict divided into: reasoning, verbal aggression, and violence [18]. For this study, 9 items from the Conflict Tactics Scale, and 19 items were chosen from the Rusbult Problem Solving Scale were selected, excluding extreme behaviors. The scale was arranged in order of severity to examine differences in problem-solving behavior between pseudo-seizures, epileptic patients, and non-epileptic patients, linking results to attachment style and personality assessment. The abuse behavior, positive behavior, and negative behavior categories were calculated based on specific item sums, with higher scores indicating more abusive (36–70), positive (26–49) or negative behavior (40–77). The scales were administered to different patient groups using validated Arabic versions of the tests.

For qualitative data, descriptive statistics were reported in frequencies and percentages. The chi-square test was employed for categorical variables, such as gender, to assess associations. An unpaired Student’s t-test was used for continuous variables, e.g., age, to compare means between groups. All statistical tests were two-tailed, and a significance level of ≤0.05 was considered statistically significant. The statistical analyses were performed using SPSS software version 22.0 (IBM, Armonk, NY, USA).

## 3. Results

This study enrolled a total of 68 participants, of which 46 were female. The average age of the participants was 29.8 ± 9.4 years. The sample consisted of 25 individuals (36.2%) with PNES, 32 (46.4%) individuals with epilepsy, and 11 healthy controls (15.9%). Table 1 demonstrates the sample’s sociodemographic and clinical characteristics. The PNES and epilepsy groups both included an equal number of female individuals (20; 29.4% each). In contrast, only 5 male individuals with PNES and 12 male individuals with epilepsy were recruited. Participants diagnosed with PNES had an average age of 32 ± 11 years, with an age range spanning 18 to 57 years. There were no substantial differences in sociodemographic characteristics observed among the PNES, epilepsy, and healthy control groups. The intelligence quotient (IQ) of patients with PNES (88.8 ± 13.6) was lower compared to healthy controls (103.5 ± 28.0) but higher than epilepsy patients (84.6 ± 12.9).

**Figure 1:**
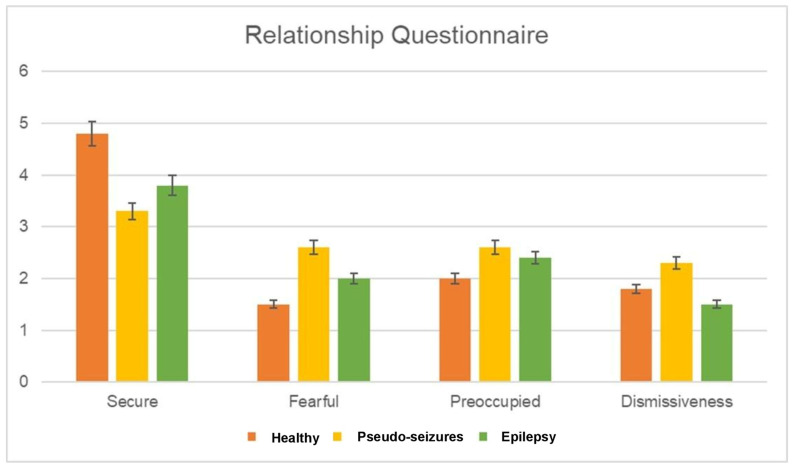
The mean scores of pseudo-seizures patients, epilepsy patients, and the healthy group for the four items (secure, fearful, preoccupied, and dismissiveness) of the Relationship Questionnaire.

Scores of the four-item RQ are presented in Figure 1 and Table 2. The PNES group had the lowest mean score (3.3 ± 1.5, *p* = 0.113) on the secure item compared to the epilepsy (3.8 ± 1.1) and healthy control groups (4.8 ± 0.5). In addition, PNES patients had higher mean scores than epilepsy and healthy controls on the preoccupied (2.6 ± 1.4 vs. 2.4 ± 1.2 vs. 2.0 ± 0.8; *p* = 0.743), fearful (2.6 ± 1.4 vs. 2.0 ± 1.2 vs. 1.5 ± 0.6; *p* = 0.312) and dismissing (2.3 ± 1.3 vs. 1.5 ± 0.7 vs. 1.8 ± 1.0; *p* = 0.234) items of the RQ. However, there were no significant differences in the scores for different attachment styles (secure, fearful, preoccupied, dismissive) among the groups (*p* > 0.05). On the RAS, PNES patients had low scores in comparison to epilepsy and healthy controls (58.8 ± 20.8 vs. 76.6 ± 10.2 vs. 72.5 ± 17.5; *p* = 0.1) but differences were not significant among the groups (*p* = 0.1).

PNES patients reported lower scores compared to patients with epilepsy and healthy controls on extraversion (13.96 ± 3.46 vs. 15.17 ± 2.38 vs. 14.30 ± 1.83; *p* = 0.504), conscientiousness (14.52 ± 2.74 vs. 14.72 ± 2.31 vs. 16.70 ± 3.33; *p* = 0.150), openness (11.6 ± 2.24 vs. 13.66 ± 3.20 vs. 13.40 ± 2.72; *p* = 0.009), and agreeableness (8.6 ± 1.96 vs. 10.59 ± 3.39 vs. 8.20 ± 1.99; *p* = 0.017). Significant differences were found in agreeableness (*p* = 0.017) and openness (*p* = 0.009) among the groups. Epilepsy patients scored higher in agreeableness, while the healthy group scored higher in openness. PNES patients had a higher mean neuroticism score (13.48 ± 3.40 vs. 14.21 ± 2.06 vs. 12.70 ± 3.83; *p* = 0.599).

Participants with PNES had a comparatively lower mean score on life satisfaction than the epilepsy patients and the control group (15.2 ± 4.9 vs. 16.6 ± 4.4 vs. 16.9 ± 3.2; *p* = 0.427). However, no significant differences were observed in the total satisfaction scores among the groups (*p* > 0.05). For the CBS, PNES patients showed more negative behavior compared to epilepsy patients and the control group (37.79 ± 13.1 vs. 22 ± 11.07 vs. 28.25 ± 5.56; *p* = 0.009). Similarly, PNES patients scored higher on abusive behavior compared to the epilepsy patients and the control group (16.93 ± 5.48 vs. 15 ± 6.42 vs. 14 ± 2.94; *p* = 0.567). However, the epilepsy group scored lower on positive behaviors compared to the PNES and control groups (19.73 ± 9.77 vs. 29.36 ± 6.39 vs. 29.75 ± 4.57; *p* = 0.011). Epilepsy patients scored lower in negative behaviors (22 ± 11.07) toward themselves compared to the other groups (37.79 ± 13.1 vs. 28.25 ± 5.56). The pseudo-seizures group exhibited higher scores in Negative—Own (*p* = 0.009), Positive—Own (*p* = 0.011), Negative—Partner (*p* = 0.011), and Positive—Partner (*p* = 0.028) compared to the epilepsy and healthy control groups. The CBS partner scores also showed higher scores on negative (37.29 ± 14.37; *p* = 0.011) and abusive (23.07 ± 17.87; *p* = 0.210) behaviors. In addition, the partners of the epilepsy patients also reported that the epilepsy patients have less positive behavior (20 ± 11.19; *p* = 0.028). There were no significant differences in Abusive—Own and Abusive—Partner scores (*p* > 0.05).

## 4. Discussion

The present study explored the attachment patterns and personality traits of individuals with psychogenic non-epileptic seizure (PNES). The study demonstrated that PNES patients exhibited a higher prevalence of insecure attachment patterns, including fearful, preoccupied, and dismissing attachment styles, indicating negative feelings about themselves and others. These findings are consistent with previous research by Dagan et al. [19] and Holman et al. [20], which highlighted the significant association between insecure attachments and PNES, particularly fearful attachment, abuse, and neglect [19,20].

Previous studies on marital status have shown that individuals with epilepsy were more likely to remain single or experience divorced compared to non-epileptic patients [21,22]. Higher rates of marital discord among epilepsy patients compared to non-epileptic individuals highlight the importance of addressing mood disorders and providing therapeutic support to enhancing marital adjustment [23]. In our study, PNES patients reported lower satisfaction with their marital status, indicating potential challenges in their relationships.

Our study found that PNES patients had more insecure attachment representations compared to epilepsy patients and healthy controls. Additionally, PNES patients also reported higher rates of neglect, trauma, and lower quality of life. In line with this, previous research indicates a higher prevalence of insecure attachment in PNES patients from the UK [7,24]. Holman, Kirkby, Duncan, and Brown [20] determined that British patients with PNES exhibited fearful attachment as their primary attachment style. Moreover, the PNES group in Germany demonstrated considerably less secured and more unresolved/disorganized attachment classifications [25]. However, the existing literature presents some inconsistency in findings regarding attachment insecurity between PNES and epilepsy patients [26].

In our study utilizing the BFI, individuals with PNES patients exhibited lower in extraversion, consciousness, agreeableness, and openness compared to findings from a study conducted with South African PNES patients [8]. The observed lower extraversion in our study suggests potential social isolation and stigma associated with PNES, similar to experiences reported by Indian PNES patients [27]. A correlation was identified between increased perceived stigma associated with epilepsy and higher levels of neuroticism and lower levels of extraversion in epilepsy patients [28], similar to the findings in our study. This association was further linked to decreased social well-being. Rassart, Luyckx, Verdyck, Mijnster, and Mark [28] also reported that higher neuroticism and lower agreeableness are associated with epilepsy seizure severity and poorer health-related quality of life in PNES patients from the Netherlands. Neuroticism, characterized by feelings of worry, anxiety, fear, rage, and frustration, was higher in our PNES cohort compared to the control group [3]. This suggest that individuals with PNES may experience anxiety and emotional distress, potentially stemming from the challenges of living with a chronic and unpredictable condition beyond their control [27]. Cragar et al. [29] proposed three personality clusters for PNES patients: (1) very high neuroticism, low extraversion, low openness, high agreeableness, and low conscientiousness; (2) average on all domains; (3) very high neuroticism, average extraversion, low openness, low agreeableness, and average conscientiousness. Our findings align with cluster 1, i.e., low extraversion, low conscientiousness, low openness, low agreeableness, and high neuroticism. Therefore, our study and findings from various countries suggest that PNES patients exhibited insecure attachment patterns, lower satisfaction with marital status, higher rates of neglect and trauma, lower extraversion, consciousness, agreeableness, and openness [27,28]. However, there are some variations in the prevalence of attachment patterns and personality traits among PNES patients across different populations. Further research could investigate the psychological processes, cognitive factors, or interpersonal dynamics that mediate or moderate this relationship.

Patients with PNES share more similarities than differences across the borders and between cultures in terms of their demographic and clinical profiles. A study comparing clinical characteristics in young PNES patients from Iran, Saudi Arabia, and Canada found similar sociodemographic characteristics, clinical features, and associated factors among patients from these nations [30]. Despite these overarching similarities, there are nuances influenced by cultural, ethnic, and religious factors that may shape the clinical presentation and manifestations of PNES. Adverse experiences, such as sexual or physical abuse, showed a significant association with female PNES patients from Iran, the USA, Canada, Brazil, Argentina, and Venezuela [31]. While Western studies consistently identified a high prevalence of sexual abuse experience, ranging from 24% to 67%, as a significantly contributing factor in PNES [5,32], a study in Iran reported a comparatively lower incidence of 8.3% for sexual abuse experiences among their PNES cases [33]. This highlights potential cultural variations in the prevalence of specific contributing factors to PNES. Moreover, race and sex play a role in the associated factors of PNES, such as the significantly higher probability of being female (*p* < 0.05), being younger in age, belonging to Hawaiian or other Pacific Islander ethnic group, and having a suburban background [34]. An international study on patients from Iran, Brazil, Venezuela, and Argentina further demonstrate that PNES exhibit more similarities than differences across various cultures, emphasizing the need for the comprehensive understanding of cross-cultural influences on PNES [35]. Cultural, ethnic, and religious factors may contribute to the diverse perception and interpretations of PNES symptoms. For instance, an Indian study reported that 53.5% of PNES patients believes that PNES symptoms had been possessed by either God, ghosts, or evil, while 87% consulted faith healers for treatment [36]. This underscores the importance of exploring cultural factors to comprehend the prevalence and presentations of PNES. Comparative studies examining diverse cultural and religious contexts could provide valuable insights into the cultural and societal influences on attachment, personality, and the development of PNES. The management of PNES should adopt a sensitive and collaborative approach, considering the socio-cultural context. Additionally, the assessment of personality traits should be incorporated into the evaluation process for patients presenting with PNES [37].

Our findings emphasize the need for psychological interventions to address the specific psychological profiles observed in individuals with PNES. Psychological interventions hold promise as potential approaches for reducing the frequency of seizures in PNES patients [38]. While cognitive (behavioral) therapy has demonstrated significant effects, the choice of treatment should account for individual coping mechanisms, anger management, and attitude [1]. Psychiatric follow-up is essential, emphasizing the importance of addressing the emotional and psychological aspects of PNES, particularly the influence of stress and emotions on behavior [39]. Clinical judgment plays a pivotal role for psychiatrists and psychologists to decide effective interventions for treating or managing PNES. Additionally, adopting a client-centered or flexible approach, compared to a manualized methodology, has been suggested to potentially yield better results [7,38]. The relationship between specific PNES symptoms and distinct psychopathologies remains unknown. Therefore, for therapeutic interventions to be effective, they must be designed to accommodate a wide variety of psychiatric histories, interpersonal issues, and functional levels [40]. This emphasizes the importance of a holistic and individualized approach to psychological interventions for PNES.

Our study has several limitations that should be acknowledged. For instance, we found IQ differences between the groups, with both epilepsy and PNES patients showing lower IQ scores compared to healthy controls, a result consistent with the existing literature [41,42,43]. While the primary focus of our study was on personality and relationship dynamics, we recognize that IQ may influence these factors. To address this, we plan to include IQ as a covariate in future analyses or discuss its potential role as a confounding variable in interpreting the results. This approach will help clarify whether the observed differences in personality and relationship patterns are independent of IQ. Additionally, medication history [44] and seizure frequency/severity/duration [45] could potentially influence personality and relationship dynamics in both PNES and epilepsy patients. Unfortunately, we did not explore these variables in the current study, and we acknowledge this as a limitation. Future research will examine these factors as potential moderators to provide a more comprehensive understanding of the relationships between anti-seizure medication, seizure severity, and personality dynamics. Other limitations include the small sample size, the unequal distribution of patient groups, and insufficient control for confounding variables. These limitations were beyond the authors’ control but are important to note. As this is a pilot study, the findings offer preliminary insights into the personality and relationship dynamics of PNES patients. Future studies will address these limitations by increasing the sample size, improving control for potential confounders, and providing a more thorough analysis. These efforts will help validate and expand upon the current findings in a more robust manner. Finally, we did not assess anxiety and depression levels in the three study groups. We acknowledge that including these measures would have provided valuable data, particularly in understanding the psychological factors influencing personality and relationship dynamics in the participants. We recognize the importance of these measures and plan to incorporate them in future studies to better explore their impact on PNES, epilepsy, and healthy control groups.

## 5. Conclusions

In our single-centered cross-sectional study in Saudi Arabia, we aimed to address existing paucity of reports on the outcomes of normal personality traits and marital attachment patterns in patients with PNES. Our findings provide encouraging results on the levels of conscientiousness comparable to those found in other centers around the world. However, our study contributes to the existing literature by providing different insights into attachment patterns, marital satisfaction, and personality traits in Saudi patients with PNES. This study revealed that individuals with PNES in this region exhibit insecure attachment patterns, lower satisfaction with marital status, and distinct personality traits. Greater levels of neuroticism and lower levels of conscientiousness in patients with PNES, more anxiety disorders in PNES, and differences in the type of trauma and timing in relation to critical developmental periods were observed. These findings may provide insight into the diverse physical manifestations of PNES depending on psychological profiles which vary among regions. However, the findings should be interpreted with caution, given the limitations of the research. Further studies are needed to gain a more comprehensive understanding of the impact of psychiatric comorbidities, history of abuse, and normal personality traits in PNES patients from the Eastern Mediterranean region. Moreover, this study is significant for understanding the psychological profiles of Saudi PNES patients, informing the development of targeted interventions, and highlighting the need for further research to explore the cultural and regional variations in attachment patterns and personality traits among PNES patients.

## Figures and Tables

**Table 1:** Summary table of sociodemographic characteristics of the study population.

Socio-Demographics	Variables	Groups Based on Diagnosis			Total (%)
		PNES	Epilepsy	Healthy	
Sex	Male	5 (7.3%)	12 (17.6%)	5 (7.3%)	22 (32.3%)
	Female	20 (29.4%)	20 (29.4%)	6 (8.8%)	46 (67.7%)
	Total	25 (36.7%)	32 (47%)	11 (16.1%)	68
Age	Mean ± S.D. years	32 ± 11 years	28 ± 9 years	28 ± 6 years	29.8 ± 9.4 years
	Median	31	27	27	27
	Range	18–57	18–59	23–42	18–59
Marital status	Single	10 (14.7%)	19 (27.9%)	7 (10.2%)	36 (53%)
	Married	14 (20.5%)	11 (16.1%)	4 (5.8%)	29 (42.6%)
	Divorced	1 (1.4%)	2 (2.9%)	0 (0.0%)	3 (4.4%)
	Widow	0 (0.0%)	0 (0.0%)	0 (0.0%)	0 (0.0%)
Education level	Below University education	12 (17.6%)	19 (27.9%)	0 (0.0%)	31 (45.6%)
	Graduate	11 (16.1%)	11 (16.1%)	10 (14.7%)	32 (47%)
	Post graduate	2 (2.9%)	2 (2.9%)	1 (1.4%)	5 (7.4%)

**Table 2:** Mean ± standard deviation scores of Relationship Questionnaire (RQ), Big Five Inventory (BFI), Relationship Assessment Scale (RAS), Satisfaction with Life Scale (SWLS), and Conflict Behavior Scale (CBS) of the pseudo-seizures group, epilepsy patients, and control group.

Classifications of Psychological Evaluation		Groups Based on Diagnosis			*p*-Value
		PNES	Epilepsy	Healthy	
RQ	1 = Secure	3.3 ± 1.5	3.8 ± 1.1	4.8 ± 0.5	0.113
	2 = Fearful	2.6 ± 1.4	2 ± 1.2	1.5 ± 0.6	0.312
	3 = Preoccupied	2.6 ± 1.4	2.4 ± 1.2	2 ± 0.8	0.743
	4 = Dismissing	2.3 ± 1.3	1.5 ± 0.7	1.8 ± 1	0.234
RAS	RASTOTAL	58.8 ± 20.8	76.6 ± 10.2	72.5 ± 17.5	0.1
BFI	Extraversion	13.96 ± 3.46	15.17 ± 2.38	14.3 ± 1.83	0.504
	Agreeableness	8.6 ± 1.96	10.59 ± 3.39	8.2 ± 1.99	0.017
	Consciousness	14.52 ± 2.74	14.72 ± 2.31	16.7 ± 3.33	0.150
	Neuroticism	13.48 ± 3.4	14.21 ± 2.06	12.7 ± 3.83	0.599
	Openness	11.6 ± 2.24	13.66 ± 3.2	13.4 ± 2.72	0.009
SWSL	Total score	15.2 ± 4.9	16.6 ± 4.4	16.9 ± 3.2	0.427
CBS	Neg_Own	37.79 ± 13.1	22 ± 11.07	28.25 ± 5.56	0.009
	Pos_Own	29.36 ± 6.39	19.73 ± 9.77	29.75 ± 4.57	0.011
	Abusive_Own	16.93 ± 5.48	15 ± 6.42	14 ± 2.94	0.567
	Neg_P	37.29 ± 14.37	21.45 ± 10.05	26.25 ± 6.45	0.011
	Pos_P	28.93 ± 7.08	20 ± 11.19	31.75 ± 6.99	0.028
	Abusive_P	23.07 ± 17.87	12.18 ± 2.75	16.25 ± 0.5	0.21

## Data Availability

The datasets used and/or analyzed during the current study are available from the corresponding author on reasonable request.
